# Cost-of-illness study in a retrospective cohort of patients with dementia in Lima, Peru

**DOI:** 10.1590/S1980-57642015DN91000006

**Published:** 2015

**Authors:** Nilton Custodio, David Lira, Eder Herrera-Perez, Liza Nuñez del Prado, José Parodi, Erik Guevara-Silva, Sheila Castro-Suarez, Rosa Montesinos

**Affiliations:** 1 Servicio de Neurología, Clínica Internacional, Lima, Peru.; 2 Unidad de Diagnóstico de Deterioro Cognitivo y Prevención de Demencia, Instituto Peruano de Neurociencias, Lima, Peru.; 3 Unidad de Investigación, Instituto Peruano de Neurociencias, Lima, Peru.; 4 Unidad de Diseño y Elaboración de Proyectos de Investigación, Lima, Peru.; 5 Centro de Investigación para el Desarrollo Integral y Sostenible (CIDIS), Universidad Peruana Cayetano Heredia, Lima, Peru.; 6 Servicio de Neurología. Instituto Peruano de Neurociencias, Lima, Peru.; 7 Centro de Investigación del Envejecimiento, Facultad de Medicina Humana, Universidad San Martín de Porres, Lima, Peru.; 8 Departamento de Medicina, Hospital San Juan de Lurigancho, Lima, Peru.; 9 Servicio de Neurología de la Conducta, Instituto Nacional de Ciencias Neurológicas, Lima, Peru.; 10 Servicio de Medicina Física y Rehabilitación, Clínica Internacional, Lima, Peru.

**Keywords:** dementia, costs and cost analysis, cost of illness, health care costs, health expenditures

## Abstract

Dementia is a major cause of dependency and disability among older persons, and imposes huge economic burdens. Only a few cost-of-illness studies for dementia have been carried out in middle and low-income countries.

**Objective:**

The aim of this study was to analyze costs of dementia in demented patients of a private clinic in Lima, Peru.

**Methods.:**

We performed a retrospective, cohort, 3-month study by extracting information from medical records of demented patients to assess the use of both healthcare and non-healthcare resources. The total costs of the disease were broken down into direct (medical and social care costs) and indirect costs (informal care costs).

**Results.:**

In 136 outpatients, we observed that while half of non-demented patients had total care costs of less than US$ 23 over three months, demented patients had costs of US$ 1500 or over (and more than US$ 1860 for frontotemporal dementia). In our study, the monthly cost of a demented patient (US$ 570) was 2.5 times higher than the minimum wage (legal minimum monthly wage in Peru for 2011: US$ 222.22).

**Conclusion.:**

Dementia constitutes a socioeconomic problem even in developing countries, since patients involve high healthcare and non-healthcare costs, with the costs being especially high for the patient's family.

## Abstract (pt)

Demência é uma das principais causas de dependência e incapacidade entre idosos, e impõe enormes encargos econômicos. Apenas alguns estudos de custo-de-doença para a demência foram realizados em países de renda média e baixa.

**Objetivo:**

O objetivo deste estudo foi analisar os custos relacionados com demência em pacientes de uma clínica particular, em Lima, Peru.

**Métodos:**

Foi realizado estudo retrospectivo de uma coorte durante três meses para extrair informações de prontuários de pacientes com demência para avaliar a utilização de recursos tanto de saúde como outros. Os custos totais da doença foram divididos em diretos (despesas de assistência médica e social) e indiretos (custos de cuidados informais).

**Resultados:**

Em 136 pacientes ambulatoriais, observou-se que, enquanto metade dos pacientes não dementes teve custos totais de cuidados de menos de US$ 23 ao longo de três meses, os pacientes dementes tiveram custos de US$ 1500 ou mais (e mais de US$ 1.860 para a demência frontotemporal). Em nosso estudo, o custo mensal de um paciente demente (US$ 570) foi 2,5 vezes maior do que o salário mínimo (salário mínimo mensal no Peru em 2011: US$ 222,22).

**Conclusão:**

Demência constitui um problema socioeconômico, mesmo nos países em desenvolvimento, uma vez que os pacientes envolvem altos custos de saúde e não de saúde, com os custos sendo especialmente altos para a família do paciente.

## INTRODUCTION

The global prevalence of dementia for 2010 was estimated at 8.5% in the elderly aged 60 and over in Latin America (LA).^1^ According to projections, LA will experience a particularly rapid increase in prevalence rates in the coming years.^1^ In the Andean region alone, an estimated 250,000 people were living with dementia.^1^ We previously reported a prevalence of dementia of 6.85% among elderly aged 65 years or older from Lima's urban community,^2^ thus a total of thirty thousand persons with dementia could be expected in Lima province alone.

Dementia is one of the major causes of dependency and disability among older persons,^3^ and represents a substantial health^4^ and economic^5^ burden. Dementia is devastating not only for individuals who have it, but also for their families and caregivers.^1^ The huge cost of the disease will pose a challenge to health systems in dealing with the predicted future increase in prevalence. Costs are estimated at US$ 604 billion per year at present and are set to increase even more quickly than prevalence.^5^

There is a growing body of evidence on the global economic cost related to dementia. The consensus is that dementia is already imposing huge economic burdens.^1^ However, to date, most cost-of-illness studies for dementia have been carried out in high-income countries.^6-12^ Evidence is also emerging in middle and low-income countries (MLIC),^13-17^ but as yet data remains sparse, particularly in the LA region.

A clearer understanding of the costs of dementia, and how these impact families, governments and their health and social care systems, is fundamental for raising awareness, achieving proper prioritization, and focusing efforts toward improving the lives of people with dementia and of their caregivers.

The aim of this cost-of-illness study was to analyze total, direct and indirect costs of dementia in a population of demented patients from a private clinic in Lima, Peru.

## METHODS

**Study design.** A retrospective, cohort, 3-month study was carried out during the period spanning from January 2012 to April 2013 in demented outpatients of the International Clinic in Lima, Peru.

The construction of this cohort was possible owing to the use of a dementia protocol ensuring a standardized approach for managing demented outpatients in our clinic. Based on this protocol, the evolution of the disease was assessed at definitive diagnosis and again three months later, with an additional visit whenever necessary for adjustments to patient's therapy. These evaluations included routine evaluation of both patients and their caregivers (i.e. people providing the patient with hours of care and helping them with activities of daily living). Overall evaluations are detailed in the Procedures section.

**Figure 1:**
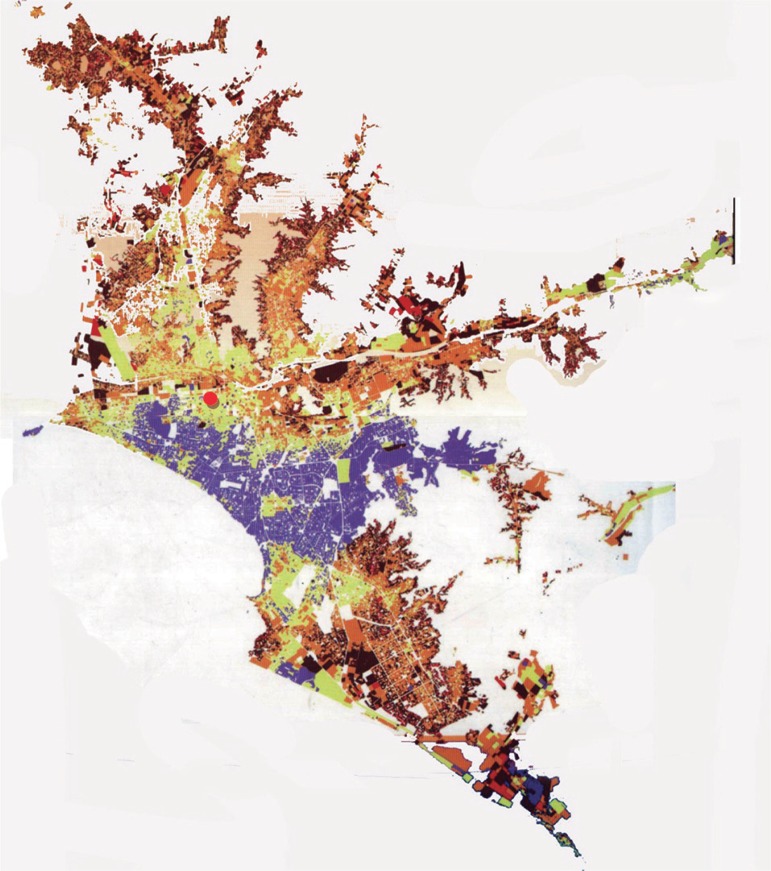
Distribution map of poverty in Lima City, Peru. The colors refer to level of poverty as following: very high incomes (purple); high incomes (yellow); middle incomes (orange); low incomes (brown); and very low incomes (red). The red circle indicates the location of the International Clinic.

**Setting and population.** The International Clinic is a private, secondary health care center in the city of Lima, capital of Peru. The clinic is located in the central region of the city, a strategic location for arrival of patients from any area of Lima. Given the catchment area, the majority of patients came from middle income areas [Figure 1].

## Procedures

*Clinical measurement scales.* The following functional measurement scales were applied:

[1] the Clinical Dementia Rating (CDR) scale;^18^
[2] the Mini-Mental State Examination (MMSE);^19,20^
[3] the Pfeffer Functional Activities Questionnaire (PFAQ);^21^ the Neuropsychiatric Inventory (NPI);^22^
[4] the Beck Depression Inventory (BDI);^23^ and
[5] the Zarit Burden Scale (ZBS).^24^

*Care services utilization.* An assessment was carried out on the use of both healthcare and non-healthcare resources (i.e. caregiver services). This was performed by extracting information from medical records. The information about non-health resources was obtained by interviewing the patient's caregiver, and this information was appended to the patient's medical record.

*Costs of dementia.* The costs of dementia are the value of all goods and services that are given up to prevent, diagnose, treat and otherwise cope with dementia. The total costs of the disease were broken down into direct and indirect costs.^16^ These costs are based on the value of resources used and resources lost, respectively.^5^

Direct costs refer to money used in an explicit way (explicitly exchanged) on drugs, hospitals, and a variety of medical or social services,^16^ differentiating medical from social care costs. While direct medical costs encompass the medical care system (such as costs of hospital care, drugs and visits to clinics), direct social costs arise from formal services provided outside of the medical care system (such as home care, food supply, transport, and residential or nursing home care).^5^

On other hand, indirect costs generally refer to productivity losses associated with the illness. Because these costs arise from impaired productivity (while working, sick leave, early retirement, or death) this type of indirect cost is generally less relevant in the context of dementia, where most patients are elderly (who are retired in most cases ). An additional component of these costs is the informal care costs, which arise from the unpaid inputs of family caregivers, friends and others.^5^ This concept refers to money used in an implicit way as loss of income by the patient and/or loss or reduction of income by family members or caregivers, namely hours without monetary reimbursement that informal caregiver spend with patients.^16^

*Selection of patients.* Eligible patients were subjects over 60 years old, with a diagnosis of dementia according to DSM-IV-TR^25^ and based on the neurologic and neuropsychological assessment. Those patients with limited functional independence and without a reliable and consistent caregiver were excluded. For the "control" group (non-demented subjects), relatives of dementia patients who had no dementia symptoms and completed the protocol with negative results for dementia diagnosis were recruited.

Dementia subtypes were defined according to internationally accepted criteria:

[1] NINCDS-ADRDA criteria^26^ for probable dementia of Alzheimer-type;
[2] Lund and Manchester criteria^27^ for frontotemporal dementia; and
[3] NINDS AIREN criteria for probable vascular dementia.^28^

*Data collection.* Data were collected through a report form designed for this study, and extracted from medical records. The following data were collected:

[1] demographics (age, sex and education);
[2] administrative (healthcare system subtype at the clinic);
[3] clinical; and
[4] care services.

*Clinical data.* This data group included medical history of dementia, treatments for dementia management, and clinical status of patient or caregiver as measured by the previously mentioned scales (CDR, MMSE, PFAQ, NPI, BDI, and ZBS).

*Care services data.* This data group included information regarding utilization of healthcare and non-healthcare resources, collected by extracting information from medical records.

*Cost estimation.* The time horizon of the study was three months, and 2013 prices were used for cost calculations. Overall costs were calculated by multiplying unit cost by resources consumption during the 3-month follow-up. The costs per person were separated according to resource consumption: diagnosis or management of dementia. Cost per patient due to dementia management, both overall and per component, was calculated using the total three-monthly costs as following:

**Total Costs** = (Medical Care Costs) + (Social Care Costs) + (Informal Care Costs)

The replacement cost approach was used to estimate indirect costs, based on the legal minimum monthly wage in Peru for 2011 (US$ 222.22). This proxy was used only for informal caregivers aged 15 to 64 years old (i.e. economically active population, EAP). For projection purposes, only costs of managing dementia were used for calculating (thus, costs of diagnosing dementia were excluded) Total Variable Costs.

To allow comparisons between countries, costs were expressed as US dollars, converted from local currencies based on exchange rates during study execution (average exchange rate in 2013 was 1 US dollar = 2.7 Peruvian "nuevo sol").

**Data analysis.** Descriptive statistics were applied to all variables, including measures of frequency distribution for qualitative variables, as well as central tendency (mean or median) and of statistical variability (standard deviation or minimum and maximum values) for quantitative variables. The Kolmogorov-Smirnov test was applied to test normality assumption of variables.

Differences among dementia subtypes were estimated using ANOVA (for Gaussian data) and Kruskal-Wallis (for non-Gaussian data) tests for quantitative variables. Post-hoc Bonferroni tests and post-hoc Mann-Whitney tests were used as appropriate. Qualitative variables were evaluated using the Chi square test (χ^2^-test).

The factors assessed (characteristics of patient and caregiver) were used for multivariate analysis through a linear regression model using "total variable costs over three months" as the dependent variable for the explanatory analysis. Because the costs variable shows a skewed right distribution, estimation was also done with its natural log (log-transformed variable total costs). This analysis was also applied to dementia subtypes. The stepwise selection method was employed (backward hierarchical selection) to identify the best model, using 0.20 as the significance level for removal from the model. This analysis was carried out with an exploratory purpose, using adjusted R squared to estimate the predictive power of the statistics models selected.

In all analyses, p<0.05was considered significant. Results were treated using the statistical software package STATA, version 12.0 (StataCorp LP College Station, TX, USA).

**Ethics statement.** Written informed consent was obtained from participants (or their relative in the case of dependent patients) and their clinical records were used for this study. The information on patients was anonymized and de-identified prior to analysis. The study protocol was approved by the local research ethics committee of the Universidad San Martín de Porres of Lima, Peru.

## RESULTS

In the present study, clinical records were collected from 136 outpatients with complete data. All selected patient with dementia had at least one caregiver. The unit costs are shown in Table 1.

**Table 1:** List of unit costs of health products or services according to healthcare delivery system at the Clinic “Internacional” in Lima, Peru.

Expenditure		Any health provider	“Family Health” program	None	Any healthcare system
Medical tests at baseline*		NA	NA	NA	186.90
Radiographic studies	Brain CT	NA	NA	NA	184.60
	Brain MRI	NA	NA	NA	250.00
Drugs	Exelon 10	NA	NA	NA	6.20
	Reminyl 16 ER	NA	NA	NA	8.90
	Donepezil 10	NA	NA	NA	3.50
	Memantine 10	NA	NA	NA	1.60
	Risperidone 2	NA	NA	NA	3.70
	Quetiapine 100	NA	NA	NA	3.50
	Olanzapine 10	NA	NA	NA	11.10
	Clonazepam 2	NA	NA	NA	1.03
	Alprazolam 0.5	NA	NA	NA	1.03
	Bromazepam 3	NA	NA	NA	0.77
	Carbamazepine 200	NA	NA	NA	0.37
	Valproate 500	NA	NA	NA	1.26
	Sertraline 50	NA	NA	NA	3.10
	Escitalopram 10	NA	NA	NA	3.60
Medical visit		37.04	18.52	11.11	NA
Hospitalization per day	Neurology service	211.50	173.10	134.60	NA
	Intensive care unit	365.40	326.90	288.50	NA

* Medical tests at baseline include: CBC, TSH, T4, GOT, GPT, glucose, urea, creatinine, electrolytes, vitamin B12, folic acid, ELISA for HIV, and VDRL. NA: Not-applicable.

**Demographic and clinical characteristics of patients.** Regarding patients' demographic and clinical variables, a significantly higher age was noted in patients with Alzheimer's dementia and also more years of education in patients with vascular dementia relative to other patients. However, patients with Alzheimer's dementia showed a significantly higher CDR with respect to patients with vascular dementia (Table 2).

**Table 2:** Demographic and clinical characteristics of 136 outpatients over 60 years old and their caregivers attended at the Neurology Department of Clínica Internacional in Lima, Peru.

Characteristics			Not dementia (n=30)		Alzheimer’s dementia (n=44)			Frontotemporal dementia (n=18)			Vascular dementia (n=44)	
			n (%)		n (%)	p-value		n (%)	p-value		n (%)	p-value
Patients	Age (years)*		67.13 [2.29]		71.87 [5.17]	<0.01		67.72 [3.10]	1.00**		69.09 [4.45]	0.30**
	Sex: female		19 (63.33)		29 (65.91)	0.82		10 (55.56)	0.59		24 (54.55)	0.45
	Education (years)*		10.23 [2.60]		11.91 [2.99]	0.06		11.61 [2.12]	0.56		12.32 [2.76]	<0.01
	Disease evolution (months)*		0.00		33.25 [7.91]	<0.01		29.61 [7.88]	<0.01		31.91 [9.72]	<0.01
	Clinical Dementia Rating (CDR)*		0.167 [0.24]		2.25 [0.61]	<0.01		1.94 [0.54]	<0.01		1.60 [0.66]	<0.01**
	Mini-mental State Examination*		28.53 [1.20]		22.43 [3.34]	<0.01		25.78 [1.40]	<0.01**		20.98 [2.48]	<0.01**^#^
	Pfeffer Functional Activities Questionnaire*		3.26 [0.73]		20.86 [2.76]	<0.01		19.72 [1.36]	<0.01		19.00 [3.62]	<0.01**
	Neuropsychiatric Inventory*		4.90 [2.16]		25.68 [6.52]	<0.01		32.61 [8.33]	<0.01**		17.91 [3.78]	<0.01**^#^
	Beck Depression Inventory*		4.27 [1.34]		16.18 [4.09]	<0.01		21.22 [3.02]	<0.01**		12.91 [3.83]	<0.01**^#^
Primary caregivers	Age (years)*		NA		48.75 [15.44]	NA		51.22 [13.67]	NA		50.34 [13.12]	NA
	Sex: female		NA		38 (86.36)	NA		17 (94.44)	NA		36 (81.82)	NA
	Education (years)*		NA		10.11 [2.46]	NA		9.56 [3.13]	NA		9.84 [2.78]	NA
	Relationship to patient	Spouse or partner	NA		9 (20.45)	NA		5 (27.78)	NA		15 (34.09)	NA
		Son or daughter	NA		13 (29.55)	NA		5 (27.78)	NA		13 (29.55)	NA
		Brother or sister	NA		9.09	NA		16.67	NA		50.00	NA
		Other family	NA		5 (11.36)	NA		1 (5.56)	NA		5 (11.36)	NA
		Hired caregiver	NA		12 (27.27)	NA		3 (16.67)	NA		9 (20.45)	NA
	Zarit Burden Inventory*		NA		28.39 [9.10]	NA		27.72 [6.96]	NA		31.27 [8.94]	NA

* Values expressed as mean [standard deviation];

** Statistically different with Alzheimer’s dementia;

# Statistically different with frontotemporal dementia; NA: Not -applicable.

Patients with vascular dementia and frontotemporal dementia patients had the lowest and highest MMSE scores, respectively. An inverse pattern was observed when assessing the NPI and BDI scales, where patients with Alzheimer's dementia had average values for these scales. All these differences were statistically significant. However, patients with vascular dementia showed significantly better PFAQ scores than patients with Alzheimer's dementia. No significant differences in sex or caregiver characteristics were observed (Table 2).

**Resource consumption.** A significantly higher proportion of use of magnetic resonance was found for diagnosis of frontotemporal dementia with respect to other dementia subtypes. All dementia subtypes were associated with a significantly higher number of medical visits compared to control patients. A significantly higher proportion of patients with Alzheimer's dementia needed anti-dementia drugs for symptoms control relative to other dementia subtypes, unlike the consumption pattern of anti-psychotic drugs observed. No significant differences in the consumption of non-healthcare resources were observed (Table 3).

**Table 3:** Resource consumption during three months for 136 outpatients over 60 years old attended at the Neurology Department of Clínica Internacional in Lima, Peru.

Resource item		Not dementia (n=30)		Alzheimer’s dementia (n=44)			Frontotemporal dementia (n=18)			Vascular dementia (n=44)	
		n (%)		n (%)	p-value		n (%)	p-value		n (%)	p-value
Healthcare resources	Healthcare delivery system										
	Any health provider	10 (33.33)		12 (27.27)	Ref.		4 (22.22)	Ref.		18 (40.91)	Ref.
	“Family Health” program	11 (36.67)		24 (54.55)	0.14		9 (50.00)	0.59		19 (43.18)	0.20
	None	9 (30.00)		8 (18.18)	0.64		5 (27.78)	0.69		7 (15.91)	0.19
	Medical tests at baseline										
	Hematological and tomography	30 (100.00)		11 (25.00)	Ref.		0.00	Ref.		11 (25.00)	Ref.
	Hematological and magnetic resonance	0.00		33 (75.00)	<0.01		18 (100.00)	<0.01**		33 (75.00)	<0.01
	Medical visits per trimester*	2.00 [0.00]		3.27 [0.59]	<0.01		3.28 [0.57]	<0.01		3.23 [0.52]	<0.01
	Hospitalizations per trimester*	1.00 [0.00]		1.23 [0.52]	0.22		1.22 [0.55]	0.63		1.18 [0.50]	0.57
	Antidementia drugs consumption	0.00		35 (79.55)	<0.01		8 (44.44)	<0.01**		19 (43.18)	<0.01**
	Antipsychotics drugs consumption	0.00		29 (65.91)	<0.01		18 (100.00)	<0.01**		39 (88.64)	<0.01**
Non-healthcare resources	Nappy consumption per day*	0.2 [0.61]		0.57 [1.17]	0.99		0.78 [1.31]	0.50		0.59 [1.23]	0.84
	Caregiver per trimester										
	None/caregiver non-EAP	30 (100.00)		8 (18.18)	Ref.		2 (11.11)	Ref.		6 (13.64)	Ref.
	Informal caregiver	0.00		24 (54.55)	<0.01		13 (72.22)	<0.01		29 (65.91)	<0.01
	Remunerated caregiver	0.00		12 (27.27)	<0.01		3 (16.67)	<0.01		9 (20.45)	<0.01

* Values expressed as mean [standard deviation];

** Statistically different with Alzheimer’s dementia; Ref: Reference categories; Caregiver non-EAP: caregiver aged over 64 years.

**Costs by dementia subtype.** While half of non-demented patients had total variable costs of healthcare and non-healthcare of less than US$ 23 over three months, costs for patients with dementia were US$ 1500 or over, and more than US$ 1860 for frontotemporal dementia patients in the three-month period (Table 4).

**Table 4:** Healthcare and non-healthcare costs during three months for 136 outpatients over 60 years old attended at the Neurology Department of Clínica Internacional in Lima, Peru.

Cost item		Not dementia (n=30)		Alzheimer’s dementia (n=44)			Frontotemporal dementia (n=18)			Vascular dementia (n=44)	
		p50 [min-max]		p50 [min-max]	p-value		p50 [min-max]	p-value		p50 [min-max]	p-value
Healthcare costs	Medical tests at baseline	372 [372-372]		437 [372-437]	<0.01		437 [437-437]	<0.01*		437 [372-437]	<0.01#
	Medical visits per trimester	37 [22-74]		56 [33-148]	<0.01		56 [33-185]	0.01		56 [33-111]	0.03
	Hospitalizations per trimester	0		0 [0-1519]	0.01		0 [0-1154]	0.02		0 [0-1519]	0.04
	Antidementia drugs	0		437 [0-846]	0.01		0 [0-558]	<0.01*		0 [0-801]	<0.01*
	Antipsychotics drugs	0		125 [0-1138]	<0.01		927 [324-1647]	<0.01*		227 [0-1423]	<0.01#
	Sub-total 1 (healthcare costs)	393 [372-409]		1167 [703-3487]	<0.01		1544 [849-3296]	<0.01*		908 [471-3126]	<0.01*#
Non- healthcare costs	Nappy consumption per trimester	0 [0-198]		0 [0-396]	0.18		0 [0-297]	0.07		0.00 [0-396]	0.18
	Caregiver per trimester			667 [667-1111]	<0.01		667 [667-1111]	<0.01		667 [667-1111]	<0.01
	Sub-total 2 (non-healthcare costs)	0 [0-198]		666 [0-1508]	<0.01		667 [0-1409]	0.03		667 [0-1409]	<0.01
	Total costs	394 [372-607]		1878 [715-4896]	<0.01		2252 [1397-4705]	<0.01		1727 [644-4188]	<0.01#
	Total variable costs	22 [0-235]		1470 [344-4459]	<0.01		1869 [960-4268]	<0.01*		1291 [207-3751]	<0.01#

* Statistically different with Alzheimer’s dementia;

# Statistically different with frontotemporal dementia; Ref: Reference categories; Total variable costs refer to costs of management of dementia, excluding the costs of dementia diagnosis. Costs are presented as US dollars (average exchange rate in 2012: 1 US dollar = 2.7 Peruvian nuevo sol).

A significantly higher cost was observed for diagnosis of frontotemporal dementia than for the other dementia subtypes. All dementia subtypes had significantly higher costs of medical visits and hospitalizations with respect to non-demented patients. Significantly higher costs of anti-dementia drug and anti-psychotic drug consumption were noted among patients with Alzheimer's dementia and with frontotemporal dementia, respectively, as compared to the other dementia subtypes. Regarding healthcare costs, patients with frontotemporal dementia had significantly higher costs and patients with vascular dementia significantly lower costs (Table 4).

No significant differences in non-healthcare costs were observed between dementia subtypes. Total costs were significantly higher in patients with dementia. Frontotemporal dementia was associated with higher total variable costs relative to both vascular dementia and Alzheimer's dementia (Table 4).

**Factors associated with costs of dementia.** Applying the backward hierarchical selection method revealed that the best model to explain total costs of dementia in demented patients overall included the following variables: dementia subtype, CDR and caregiver age. Also, the multivariate analysis showed that these variables were not significant for all dementia subtypes. Only CDR and caregiver age were associated with significantly higher costs in patients with Alzheimer's dementia and patients with vascular dementia. In patients with frontotemporal dementia, only hired caregiver was statistically associated with higher total variable costs (Table 5).

**Table 5:** Linear Regression models showing both the factors associated with higher total (variable) for overall dementia and by dementia subtype in 106 demented outpatients over 60 years old attended at the Neurology Department of Clínica Internacional in Lima, Peru.

Factors			Overall dementia patients (n=106)		Alzheimer’s dementia (n=44)		Frontotemporal dementia (n=18)		Vascular dementia (n=44)	
			β Coefficient	p-value	β Coefficient	p-value	β Coefficient	p-value	β Coefficient	p-value
Dementia subtype	Alzheimer’s dementia		Ref.	Ref.	NA		NA		NA	
	Frontotemporal dementia		0.43	<0.01	NA		NA		NA	
	Vascular dementia		0.15	0.19	NA		NA		NA	
Healthcare delivery system	Health provider		Ref.	Ref.	Ref.	Ref.	Ref.	Ref.	Ref.	Ref.
	“Family Health” program		–0.05	0.61	–0.04	0.84	0.07	0.70	0.05	0.84
	None		–0.21	0.06	–0.18	0.32	0.07	0.77	–0.19	0.44
Patients	Age (years)		<0.01	0.57	0.01	0.47			–<0.01	0.63
	Sex: female		–0.03	0.73	0.06	0.67			–0.23	0.20
	Education (years)		–<0.01	0.82	0.01	0.60			–0.05	0.14
	Clinical Dementia Rating (CDR)***		0.38	<0.01	–0.47	<0.01	0.06	0.75	0.40	<0.01
	Mini-mental State Examination		0.02	0.31	0.03	0.11	–0.03	0.75	–0.06	0.10
	Pfeffer Functional Activities Questionnaire		<0.01	0.80	–<0.01	0.68	–0.06	0.38	0.01	0.61
	Neuropsychiatric Inventory		<0.01	0.90	–<0.01	0.65	0.02	0.05	–<0.01	0.72
	Beck Depression Inventory		–0.02	0.13	–0.02	0.23			–0.01	0.55
	Disease evolution (months)		–0.01	0.17	–<0.01	0.67			–0.02	0.10
Primary caregivers	Age (years)		–0.02	<0.01	–0.04	<0.01			–0.02	0.02
	Education (years)								0.05	0.12
	Hired caregiver	No, family member	Ref.	Ref.	Ref.	Ref.	Ref.	Ref.	Ref.	Ref.
		Yes, hired caregiver	–0.17	0.27	–0.47	0.09	0.67	0.02	–0.26	0.34
	Adjusted R-squared		0.52		0.60		0.46		0.33	

NA: Not -applicable ; Total variable costs refer to costs of management of dementia, excluding the costs of dementia diagnosis; Empty cells (data not showed) indicate that this group of variables was removed from the model (p-value ≥0.20).

The predictive ability of these models is variable. However, the selected model explains 60% of the total variable cost variance in patients with Alzheimer's dementia (Table 5).

## DISCUSSION

This study has shown that demented patients had much higher total healthcare and non-healthcare costs than non-demented elderly. More than half of patients with frontotemporal dementia, Alzheimer's dementia, and vascular dementia, had costs of over US$ 1800, US$ 1400, and US$ 1200, respectively, during the first three-month after diagnosis. The average total monthly cost per patient with dementia during the study period was approximately US$ 570/month (US$ 6844 per year), primarily due to an elevated consumption of healthcare resources including the use of antidementia and antipsychotic drugs, an amount representing more than 2.5 times the legal minimum monthly wage in Peru for 2011. Thus, our results are in line with those previously reported in the literature.^10,15,16^

On the other hand, these costs are much lower than those seen in other countries because costs tend to be lower in developing countries, both per person and societally. This occurs because, in these regions, there is much greater reliance on unpaid informal care provided by family and others.^5^

In the present study, total costs of dementia were significantly associated with dementia subtype, CDR and caregiver age, independently of patient age, sex or years of education. Level of clinical deterioration, as evaluated with the CDR scale, was associated with a significantly higher cost for the disease, similar to that observed in other studies^29-32^ which used the Blessed Dementia Rating Scale. Thus, the total cost of the disease appears to be better explained by the greater use of both healthcare and non-healthcare resources, that may be associated with patient severity, as directly measured by the CDR scale, and indirectly by the greater need for care (leading to hiring of a caregiver). It is possible that caregiver age serves as an indicator of indirect costs owing to loss or reduction of income by family members of working age (economically active population).

The factors identified by this study differ to those of the study by Rojas et al.^15^ The cited study found CDR values to be associated with higher total cost only in patients with frontotemporal dementia. Moreover, only BDI and instrumental activities of daily living (IADL) values proved statistically associated with total costs of dementia.^15^ Since the IADL scale was not used in the present study, it was not possible to explore this association. However, no differences in PFAQ values were found.

Previous studies had not explored the utility of explanatory models by dementia subtypes, and the potentially predictive power in patients with Alzheimer's dementia (most frequent dementia subtype) is considerable. Additionally, we noted that non-healthcare costs did not differ statistically between dementia subtypes. Thus, healthcare costs are critical in the total costs of the disease. A higher rate of requests for magnetic resonance inpatients with frontotemporal dementia could be associated with greater requirements for establishing an accurate diagnosis.^33^

The costs of drugs appear to be the main resource in healthcare costs. Antidementia drug consumption was highest in patients with Alzheimer's dementia, in accordance with guideline recommendations.^34^ Support for their use in patients with frontotemporal dementia^35^ or vascular dementia^36^ is limited. By contrast, antipsychotic drug consumption was highest in patients with frontotemporal dementia, corroborating previous studies. ^37.38^ The degenerative dementias (i.e. Alzheimer's dementia and frontotemporal dementia) appear to be more expensive than vascular dementia. Our results are similar to those previously reported by Rojas et al.^15^ The cost of antidementia drugs in Peru is very high and the monthly cost of standard treatment exceeds the legal minimum monthly wage for 2013 (222.22 US dollars).

Our study did not consider the drugs or medical services for other medical conditions (such as chronic non-communicable diseases) that have a high prevalence in this age group, especially in patients with vascular risk factors or vascular dementia that require medication on a chronic basis. In the present study, no significant influence of other characteristics of the patient (age, sex, years of education, disease evolution, MMSE, PFAQ, NPI and BDI values) or of the primary caregiver (years of education and ZBI values) on total costs of disease was found. Therefore, according to the data, the total cost of dementia does not depend on these variables. Increased disease evolution time can be expected to be accompanied by, as the patient's situation worsens, a greater need for the participation of professional and paid caregivers, further increasing the total cost of the disease. However, given that only the CDR scale showed a significant difference on univariate analysis, it is noteworthy that in terms of evaluation scales, the CDR may serve as an overall indicator of other scales applied in demented patients.

Some studies in LA have found similar cost patterns for dementia, showing that indirect costs constitute the largest proportion of expenditure and almost all cost-of-illness is funded by the patient's own family.^10,16^ While in Spain the monthly overall mean cost of AD (US$ 1760.78) was almost three times the minimum wage - MW (average MW for 2003-2006: US$ 612.92), in Argentina (average MW for 2002-2008: US$ 163.62) monthly cost (US$ 644.67) were almost four times the MW. In Peru, the monthly cost of a demented patient (US$ 570) was only 2.5 times the MW. Thus, social costs appear to be even greater in developed countries. Although our study did not further evaluate indirect costs, it highlighted an interesting social phenomenon: a substantial proportion of caregivers of working age, which constitutes a social problem.

The estimated annual worldwide cost to society of dementia (costs of dementia) of US$ 604 billion in 2010, highlights the enormous impact that dementia has on socioeconomic conditions worldwide.^1^ Costs of informal care and the direct costs of social care represent similar proportions (42%) of total costs worldwide, while direct medical care costs are much lower (16%).^5^ However, our study found that almost 90% of total expenditure was dedicated to direct medical care costs. Thus, future prospective research should appropriately measure the resources associated with informal costs in demented patients and their families.

Some limitations of the present study should be highlighted:

[1] the sample size is low and the sample design used is not representative (precluding inference);
[2] with the selected population (outpatients living at home), it was not possible to obtain any information about institutionalized patients;
[3] information bias is possible since retrospective data was used;
[4] there may be other demographic (marital status, presence of cohabitants, income, living arrangements), clinical (existence of comorbidities) and caregiver (caregiver living with the patient or otherwise, time spent on care, and time spent on supervision) variables not considered in this study, as well as variables for adequately assessing the non-healthcare resources (inventory materials, swabs and dressing materials, housing structural adaptation etc.) and costs; and
[5] this study was a cohort that only covered 3 months of follow-up, precluding assessment of the dynamics of resource utilization over periods of one year or longer. We sought to estimate resources minimally expected for a period of one year using the information from one trimester

Nevertheless, we conducted a study with analytical purposes (not inferential), which constitutes the first publication reporting the factors associated with costs of dementia in a Peruvian elderly population, specifically in outpatients living at home in Lima. This study begins a line of research to be continued by prospective studies assessing costs of dementia (overall and by subtype).

In conclusion, this study found that type of health system coverage, CDR value, and hired caregiver in demented community-dwelling outpatients were associated with total costs of the disease, which proved far higher than in non-demented elderly. Thus, dementia constitutes a socioeconomic problem even in developing countries since patients involve high healthcare and non-healthcare costs, with these costs being especially great for the patient's family.

The seeking of medical help is relatively unusual in low and middle income countries, where dementia is often viewed as a normal part of ageing. Demand for medical care is likely to increase in the future, with improved awareness, posing a challenge for governments to respond to the substantial growing numbers of people with dementia. A broad public health approach is needed to improve the care and quality of life of people with dementia and their family caregivers.
